# Examining the use, confidence, and barriers to follow Advanced Life Support in Obstetrics (ALSO) course guides in managing obstetric emergencies in Sudan

**DOI:** 10.1186/s12909-024-05159-x

**Published:** 2024-02-22

**Authors:** Esra Abdallah Abdalwahed Mahgoub, Sarah H. M. Osman, Hafeia A. Al-Hussien, Nehal Al-Bushra, Amna Khairy, Yasir Ahmed Mohammed Elhadi

**Affiliations:** 1 One Percent Research Initiative, Khartoum, Sudan; 2 Faculty of Medicine, University of Khartoum, Khartoum, Sudan; 3 Al-Maali Polyclinic, Omdurman, Sudan; 4 Technical Officer, Eastern Mediterranean Region Network for Public Health, Khartoum, Sudan; 5 Department of Public Health, Sudanese Medical Research Association, Khartoum, Sudan

**Keywords:** ALSO, Obstetrics emergencies, Sudan, Confidence

## Abstract

**Background:**

The Advanced Life Support in Obstetrics (ALSO) course is a globally recognized interprofessional training program designed to assist healthcare professionals in acquiring and sustaining the necessary knowledge and skills to handle obstetric emergencies effectively. This survey aimed to assess the use, barriers, and confidence in using the ALSO course guidelines in managing obstetric emergencies in Sudan.

**Methods:**

This descriptive cross-sectional study involved 103 physicians from the Sudan ALSO group in Sudan. A structured, close-ended questionnaire was distributed electronically to the participants. Data analysis was conducted using Statistical Package of Social Sciences Software version 26.

**Results:**

More than half of the participants were specialists (54.4%). Although all respondents claimed to adhere to the ALSO guidelines for managing shoulder dystocia, a lower percentage followed them for neonatal resuscitation (75.0%) and maternal venous thrombosis management (68.9%). Only 62.1% of participants felt confident performing neonatal resuscitation. The main barriers to implementing the ALSO course guidelines were the respondents’ preference for other guidelines and their belief that the guidelines were not applicable in their specific settings.

**Conclusion:**

The majority of participants displayed a high level of confidence, indicating a positive perception of the guide's effectiveness. However, there is room for improvement, particularly in areas such as neonatal resuscitation and forceps-assisted births, where confidence levels were lower. Addressing barriers, including the preference for other guidelines and the applicability of the guide in specific settings, is crucial to ensure widespread adoption. Refresher training programs, contextual adaptations, and the integration of guidelines may help overcome these barriers and enhance the overall implementation of the ALSO guide in managing obstetric emergencies in Sudan.

## Introduction

The World Health Organization recorded the deaths of 287,000 women globally during and after pregnancy in 2020, with 95% of these deaths occurring in low and lower-middle-income countries [1]. The majority of these deaths were deemed preventable [1]. Sudan has a maternal mortality rate of 295 deaths per 100,000 live births and a neonatal mortality rate of 27 deaths per 1,000 live births, which is considered high compared to other countries in the region [2, 3]. Maternal and Child Health services in Sudan are provided by midwives at the community level and health cadres at the facility level [4], by a total of 184 hospitals and 741 health centers [5]. However, there is a clear imbalance in the distribution of health services across the nation [5]. Among the eighteen states of the country, West Kordofan, and Khartoum states had the highest number of maternal deaths [6]. According to the Sudan Maternal Death Surveillance and Response Report of 2020, 89.6% of maternal deaths in the country were attended by healthcare providers [6]. The report also highlighted the low skills and coverage of community midwives and community healthcare providers, impacting their ability to identify at-risk pregnancies and refer them to care promptly [6]. The Federal Ministry of Health in Sudan has emphasized the need to enhance the capacity of healthcare providers and community midwives in providing basic and emergency obstetric and neonatal care, leading to the importance of courses focusing on the standardized management of obstetric emergencies [6].

The Advanced Life Support in Obstetrics (ALSO) course is an interprofessional course that supports health professionals in developing and maintaining knowledge, skills, and confidence in managing obstetric emergencies. It was established by a group of family physicians in Wisconsin, UAS, in 1991 [7–9]. It has been taught outside the United States since 1995 in 62 countries. The course involves reading materials, lectures, and hands-on materials, collectively called "The ALSO Guide" of management. In Sudan, the course started in 2004 at Soba University Hospital, affiliated with the University of Khartoum [10], with more than 160 courses distributed nationwide, targeting mainly physicians, nurses, and midwives and open to all interested healthcare workers. In 2013, the Sudan ALSO group collaborated with the Sudanese Medical Specialization Board, and the course became compulsory for all obstetrics and gynecology registrars as part of their training program [10].

ALSO training improves obstetric outcomes by enhancing skills and practices and increasing confidence in handling obstetric emergencies such as postpartum hemorrhage, managing the third stage of labor, and vacuum-assisted vaginal delivery [11–13]. There was, however, a decrease in knowledge related to emergencies as early as six weeks after the course [7]. Moreover, studies have found that skills decline over time more than knowledge [14] and that training is not associated with real-life changes in providers' conduct [15–17]. In addition to the lack of refresher training opportunities, a lack of post-training supervision and guidance and obtaining enough opportunities to apply new knowledge and skills forms an issue in applying ALSO training [18].

Despite the great effort of ALSO faculty in Sudan to maintain course continuity and quality and their endeavor to spread the course around the country, there is only one published study abstract about ALSO in Sudan in 2009 [19] that focused on the change in knowledge, skills, attitude, confidence, and motivation of ALSO instructors after the course. Therefore, this study aims to provide information about the healthcare provider's practice of ALSO guides in managing obstetric emergencies, assessing confidence and barriers in using ALSO guidelines in Sudanese hospitals. Identifying problems in applying those procedures among providers during their clinical practice would provide better insight and valuable feedback, leading to identifying educational needs and anticipating obstacles to implementing solutions in future courses.

## Materials and methods

### Study design, population, and area

This descriptive cross-sectional study involved doctors from the ALSO group in Sudan. The Sudan ALSO group refers to a collective of physicians responsible for organizing and delivering the ALSO course in Sudan. Each year, the group's membership is reviewed and updated, with doctors joining based on their exceptional performance in the written and clinical exams following the ALSO provider course. Once selected, these doctors undergo training in teaching techniques and communication skills to effectively educate course candidates. The study candidates were selected from the ALSO group given that they represent ALSO candidates with the highest scores in both written and practical course exams. As a result, they are expected to derive the maximum benefit from the course. Our research involved doctors who were practicing obstetrics and gynecology at the time of the study, regardless of their professional level. Out of the 140 doctors in the ALSO group, 103 agreed to join the study (73.6%) response rate.

### Data collection

The data were obtained using an anonymous structured, self-administered questionnaire. The questionnaire was sent to the participants from the ALSO group in Sudan via WhatsApp, and it comprises three sections: the first section contained participants’ information, the second assessed the use and barriers of using ALSO course guidelines in patient management, and the third investigated the confidence in using the ALSO methods. Data were collected from the 1st to 15th of December 2022.

### Statistical analysis

The data were entered and analyzed using Statistical Package for the Social Sciences version 26. The confidence levels using ALSO guidelines were analyzed using a five-point Likert scale, and a total score was calculated (scores range from 9 to 45). A higher score indicated a higher level of confidence. An arbitrary cut of points of 50% and 75% was set to categorize the confidence score as follows: less than 50% was considered a low level of confidence, 50% to 75% was considered a moderate level of confidence, and above 75% was considered a high level of confidence. Moreover, the total ALSO usage score was calculated as follows: using the ALSO method was given 1, and not using it was given 0 for each of the nine studied emergencies. For the inferential analysis, Mann‒Whitney, Kruskal‒Wallis, and Pearson correlation tests were used to assess the association/correlation between the variables. The significance was set at a 0.05 level of alpha error.

## Results

### Characteristics of participants

The majority of participants (76.7%) were female with a mean ± (SD) age of 32.1 ± (3.7) years and 5.5 (± 3.4) years of working experience in obstetrics and gynecology. Over half (54.4%) of the participants were specialists, while more than a third (38.8%) were registrars (Table 1).

**Table 1:** Socio-demographic and professional characteristics of participants

		%(n)
Gender	Female	76.7(79)
	Male	23.3(24)
Occupation	Consultant	1.9(2)
	Specialist	54.4(56)
	Registrar	38.8(40)
	Medical Officer	3.9(4)
	House officer	1.0(1)
Age	Mean	32.1
	Std. Deviation	3.7
Years of clinical experience in obstetrics and gynecology	Mean	5.5
	Std. Deviation	3.4

### The barriers to using ALSO guides in managing obstetric emergencies

**Fig. 1:**
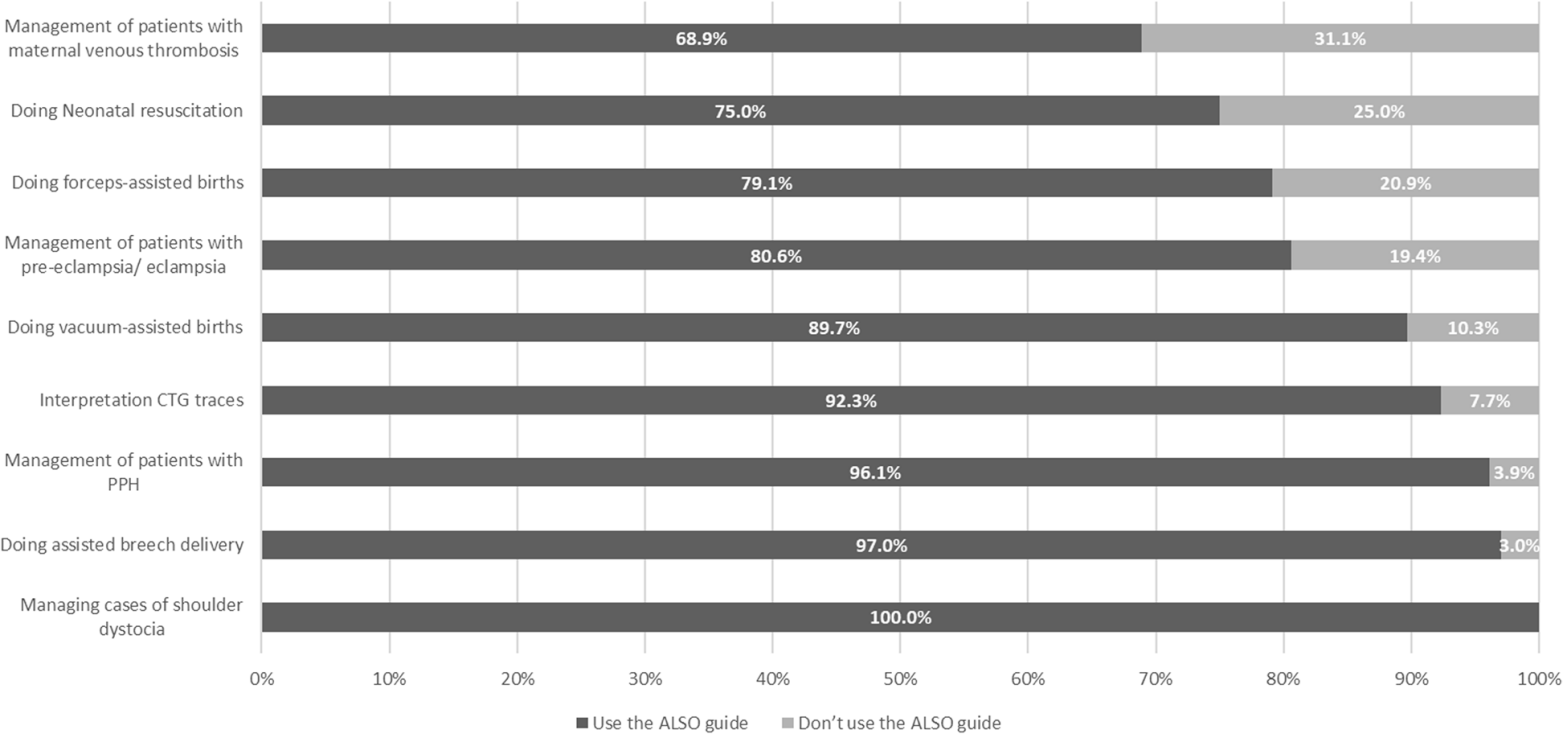
The instructor’s use of the ALSO way in managing obstetric conditions

More than 90% of the physicians in the current study utilized the ALSO guide for managing shoulder dystocia, postpartum hemorrhage (PPH), assisted breech delivery, and interpretation of cardiotocography (CTG). However, the least frequently followed aspect of the ALSO guide was in the management of maternal venous thrombosis cases (Fig. 1). Moreover, only 26.2% of the doctors in the present study used the ALSO guide to manage the nine studied obstetric emergencies, 23.3% for eight emergencies, and 16.5% for managing seven emergencies.

Regarding the barriers to implementing the ALSO guide, the most reported obstacle was the preference of doctors to follow other guidelines. This was followed by concerns about the applicability of the ALSO management method in their specific workplace and the absence of the ALSO guide as part of the hospital protocol guide (Table 2). Furthermore, there was no association between using the ALSO guide and other variables in the study.

**Table 2:** The barriers of using the ALSO way in managing obstetric emergencies

Barriers to using the ALSO guide in emergencies*	PPH*	CTG*	**Neonatal Resuscitation**	**Forceps assisted breech**	**Vacuum-assisted breech**	**Assisted breech delivery**	**Preeclampsia**	**Maternal venous thrombosis**	Total (n)
	Valid % (n)	Valid % (n)	Valid % (n)	Valid % (n)	Valid % (n)	Valid % (n)	Valid % (n)	Valid % (n)	
It is hard to remember	25.0(1)	14.3(1)	16.7(4)	-	-	-	5.0(1)	15.6(5)	10.3(12)
I prefer other ways of management (Other guidelines)	25.0(1)	42.9(3)	12.5(3)	-	22.2(2)	33.3(1)	60.0(12)	62.5(20)	35.9(42)
It is not applicable in my working setting	25.0(1)	42.9(3)	45.8(11)	27.8(5)	22.2(2)	-	5.0(1)	9.4(3)	22.2(26)
My superiors ask me to follow other guidlines of management	25.0(1)	-	8.3(2)	-	-	-	30.0(6)	12.5(4)	11.1(13)
It is usually done by paediatric specialist or resident	-	-	16.7(4)	-	-	-	-	-	3.4(4)
It is not part of the hospital protocol	-	-	-	72.2(13)	44.4(4)	66.7(2)	-	-	16.2(19)
Vacuums are not functioning	-	-	-	-	11.1(1)	-	-	-	0.9(1)
Total	100(4)	100(7)	100(24)	100(18)	100(9)	100(3)	100(20)	100(32)	100(117)

^*^Abbreviations: *ALSO* Advanced Life Support in Obstetrics, *CTG* Cardiotocography, *PPH* Postpartum Haemorrhage

### Confidence in using the ALSO guides in managing obstetric emergencies

The majority of the participants (85.4%) reported a high level of confidence in utilizing the ALSO guide for managing obstetric emergencies. In comparison, 13.5% reported a moderate confidence level, and 1% expressed a low confidence level. When examining specific obstetric emergencies, over 90% of participants expressed confidence in using the ALSO guide for managing (PPH) and preeclampsia/eclampsia cases. However, a notable proportion, 11.7% and 10.7%, respectively, lacked confidence in performing neonatal resuscitation and forceps-assisted birth (Table 3). However, there was no significant association between confidence level and participants’ characteristics (*p* > 0.05).

**Table 3:** The instructor’s confidence in using the ALSO way in managing obstetric conditions

	**Confidence level**		
	Extremely not Confident / Not Confident	Neutral	Confident / Extremely Confident
Management of patients with PPH*	2.9(3)	3.9(4)	93.2(96)
Management of Pre-eclampsia/eclampsia	1.9(2)	7.8(8)	90.3(93)
Doing assisted breech delivery	2.9(3)	7.8(8)	89.3(92)
Managing cases of shoulder dystocia	5.8(6)	6.8(7)	87.4(90)
Doing vacuum-assisted births	5.8(6)	9.7(10)	84.5(87)
Interpretation CTG traces*	3.9(4)	21.4(22)	74.8(77)
Management of Maternal venous thrombosis	4.9(5)	23.3(24)	71.8(74)
Doing forceps-assisted births	10.7(11)	18.4(19)	70.9(73)
Doing Neonatal resuscitation	11.7(12)	26.2(27)	62.1(64)

^*^Abbreviations: *CTG* Cardiotocography, *PPH* Postpartum Haemorrhage

## Discussion

The findings of this study provide valuable insights into the usage, barriers, and confidence levels associated with the implementation of the ALSO course guide for managing obstetric emergencies among ALSO course-experienced physicians in Sudan. The results highlight both positive aspects and areas that require attention and improvement.

The study revealed that over 90% of the participants reported confidence in using the ALSO guide for managing postpartum hemorrhage (PPH) and preeclampsia/eclampsia cases. This finding suggests that these specific areas are well addressed in the guide and that the participants have a firm grasp of the recommended management approaches. However, the study also identified areas where confidence levels were lower. For instance, a notable proportion of participants lacked confidence in performing neonatal resuscitation (11.7%) and forceps-assisted births (10.7%). These findings highlight the need for targeted training and education programs to improve competence and confidence in these specific areas.

Furthermore, the majority of participants reported a high level of confidence (85.4%) in using the ALSO guide for managing obstetric emergencies. This indicates a positive attitude toward the effectiveness and utility of the guide in their practice. However, it is worth noting that a small proportion of participants (1%) expressed a low confidence level, indicating a need for targeted interventions to enhance their confidence and ensure consistent implementation of the guide. Similarly, several studies revealed an improvement in healthcare providers’ confidence and comfort when dealing with emergencies after training [7, 8, 11, 13, 20–23]. However, the improvement in healthcare professionals’ confidence does not ensure an improvement in their actual practice or patient outcomes [20, 24]. Providing continuous supportive supervision to doctors may help translate their confidence into their day-to-day practice.

All doctors in our study were using the ALSO guide in managing cases of shoulder dystocia, and most expressed confidence in their ability to use this method. Equivalently, studies have shown that drills and simulation training can enhance a doctor's practice and improve the outcomes of shoulder dystocia cases [17, 20, 25]. The high utility can be explained by the fact that the teaching of shoulder dystocia in ALSO courses involves using cognitive schemes known to enhance information retention [26, 27].

The ALSO method is associated with a reduction in PPH mortality in low-resource countries [13]. Our study found that many participants used the ALSO guide to manage PPH cases. Previous research indicates that training in the ALSO method improves staff proficiency in managing PPH, with this improvement lasting for an extended period [17, 20, 28]. This finding is attributed to the emphasis on PPH management in ALSO courses in Sudan, where PPH is a leading cause of maternal death [29].

The majority (92.3%) of participants used the ALSO method for interpreting cardiotocograph traces, despite having moderate confidence. This may be because the ALSO course was their primary source of information or the most straightforward guide for interpreting CTG. The decline in confidence levels could be due to participants' precourse knowledge and expertise [7]. Intrapartum fetal surveillance training does not always improve practitioners' confidence in interpreting CTG traces [15].

The survey found that neonatal resuscitation had the lowest confidence level when using the ALSO guide. Some participants struggled to recall the guide due to its complexity or believed it should be performed by pediatric specialists or residents. Studies have shown a decline in neonatal resuscitation skills more than knowledge over time [14]. Studies have shown that improvements in neonatal resuscitation skills do not significantly affect doctors’ clinical practice [16]. The decline in confidence in neonatal resuscitation may be due to its infrequent use compared to other emergency skills [7, 20]. The participants' low to moderate confidence in performing neonatal resuscitation and interpreting CTG traces could be attributed to their complexity and difficulty remembering the multiple steps involved [7, 28]. Simplifying teaching methods with an easy-to-remember guide may boost doctors' confidence and improve real-life applications. Furthermore, additional drills may improve these skills, subsequently resulting in a positive impact on one's confidence.

The practical application of instrumental delivery in real-life situations is much more complicated than its utilization on mannequins. A study in Tanzania found that although participants successfully learned the vacuum delivery technique on mannequins during ALSO training, they did not use it before or after the training [17]. In contrast, a Honduran study showed that vacuum-assisted vaginal delivery increased after the introduction of ALSO [13]. Participants in our study showed a greater inclination toward using the ALSO method for vacuum-assisted births and were more confident utilizing it than forceps-assisted births. However, the ALSO method in instrumental delivery was not a part of their hospitals’ protocols. Thus, hospital policies must be revised to prevent unnecessary cesarean sections and attempt instrumental delivery when feasible.

Simulation-based training has been proven to enhance participants' practical skills and improve their ability to apply these skills when dealing with real-life emergencies [12, 20, 23, 30]. However, in the current study, doctors were variably able to translate ALSO guidelines into their practice, regardless of their knowledge and skills. The literature has identified several factors that could contribute to this know-do gap. These barriers include the lack of post-training supervision, lack of refresher training opportunities, staff rotation, lack of devices, and most importantly, the lack of precise details in the guidelines and mixed messages from different approaches [13, 17, 18, 31, 32]. The current study revealed that the most commonly reported barrier was the preference of participants to follow other guidelines. In the absence of a national guideline for managing obstetrics and gynecology cases in Sudan, doctors rely on international guidelines (including British guidelines and WHO guidelines) according to their preference. This suggests a potential need for the alignment and integration of different guidelines to ensure consistency and reduce confusion among healthcare professionals. Additionally, participants expressed concerns about the applicability of the ALSO guide in their specific workplace settings and the absence of the guide as part of the hospital protocol. These barriers highlight the importance of considering contextual factors when implementing guidelines and the need for creating supportive environments that facilitate adherence to the recommended practices.

The study also explored the association between participant characteristics and confidence levels. Interestingly, no significant associations were found between age, gender, occupation, years of experience, and confidence scores. Similarly, the participant's usage score, which reflects their adherence to the ALSO guide, did not show significant associations with these characteristics. This indicates that these demographic factors do not necessarily influence confidence and adherence to the guide but may be influenced by other individual or contextual factors not examined in this study.

It is essential to acknowledge the limitations of this study. The cross-sectional design limits the ability to establish causality or assess changes over time. The study was conducted with a specific group of instructors in Sudan, and the study did not include midwives and nurses, which may limit generalizability to other populations. Future research could include a larger and more diverse sample including midwives and nurses to enhance the generalizability of the findings. Additionally, qualitative research methods could be employed to better understand the underlying factors influencing confidence levels, usage patterns, and barriers to implementing the ALSO guide. Further research is needed to investigate the correlation between the duration of the course and the level of confidence, as well as the relationship between confidence level and adherence to the provided guidelines, concerning maternal and neonatal mortality rates in the specific region.

## Conclusion

In conclusion, this study contributes to understanding the usage, barriers, and confidence levels associated with implementing the ALSO guide for managing obstetric emergencies in Sudan. The majority of participants displayed a high level of confidence, indicating a positive perception of the guide's effectiveness. However, there is room for improvement, particularly in areas such as neonatal resuscitation and forceps-assisted births, where confidence levels were lower. Addressing barriers, including the preference for other guidelines and the applicability of the guide in specific settings, is crucial to ensure widespread adoption. Refresher training programs, contextual adaptations, and the integration of guidelines may help overcome these barriers and enhance the overall implementation of the ALSO guide in managing obstetric emergencies in Sudan.

## Data Availability

The datasets analyzed during the current study are available from the corresponding author on reasonable request.

## Abbreviations

ALSO: Advanced Life Support in Obstetrics
CTG: Cardiotocography
PPH: Postpartum hemorrhage
